# Stem cell-derived exosomes in the treatment of acute myocardial infarction in preclinical animal models: a meta-analysis of randomized controlled trials

**DOI:** 10.1186/s13287-022-02833-z

**Published:** 2022-04-08

**Authors:** Yan-li Zheng, Wan-da Wang, Ping-yu Cai, Feng Zheng, Yi-fan Zhou, Mei-mei Li, Jing-ru Du, Shu Lin, Hui-li Lin

**Affiliations:** 1 Department of Cardiology, The Second Affiliated Hospital of Fujian Medical University, No. 34 North Zhongshan Road, Quanzhou, 362000 Fujian Province China; 2 Department of Neurosurgery, The Second Affiliated Hospital of Fujian Medical University, Quanzhou, Fujian Province China; 3 Centre of Neurological and Metabolic Research, The Second Affiliated Hospital of Fujian Medical University, Quanzhou, Fujian Province China; 4 Diabetes and Metabolism Division, Garvan Institute of Medical Research, 384 Victoria Street, Darlinghurst, Sydney, NSW 2010 Australia

**Keywords:** Meta-analysis, Acute myocardial infarction, Animal models, Stem cell-derived exosomes

## Abstract

**Background:**

Exosomes (EXOs) derived from stem cells have become a potential new treatment for acute myocardial infarction (AMI). However, their impact is still not fully understood. Therefore, we performed this meta-analysis to systematically review the efficacy of EXOs on AMI in preclinical animal models.

**Methods:**

We searched PubMed, EMBASE, and the Web of Science from September 1, 1980 to September 1, 2021, to retrieve the studies reporting the therapeutic effects of EXOs on AMI animal models. Secondary endpoints include the fractional shortening (FS), infarct size (IS), fibrosis area (FA), the TNF-α, IL-6 and IL-10 levels, the apoptosis rate and the number of autophagic vesicles. Two authors independently screened the articles based on inclusion and exclusion criteria. All statistical analyses were conducted using Stata14.0.

**Results:**

Ten studies satisfied the inclusion criteria. Pooled analyses demonstrated that the levels of LVEF (WMD = 3.67%; 95% CI 2.28–5.07%; *P* = 0.000), FS (WMD = 3.69%; 95% CI 2.06–5.33%; *P* = 0.000), IS (WMD = −4.52%, 95% CI − 7.14 to − 1.9%; *P* = 0.001), and FA (WMD = −7.04%, 95% CI − 8.74 to − 5.34%; *P* = 0.000), TNF-α (WMD = −3.09, 95% CI − 5.47 to − 0.72; *P* = 0.011), TL-6 (WMD = −6.34, 95% CI − 11.2 to − 1.49; *P* < 0.01), TL-10 (WMD = 6.37, 95% CI 1.53–11.21; *P* = 0.01), the apoptosis rate (WMD = −8.23, 95% CI − 15.29 to − 1.17; *P* = 0.000), and the number of autophagic vesicles (WMD = −4.52, 95% CI − 7.43 to − 1.62; *P* = 0.000). Subgroup analysis showed that the EXOs were derived from HMSCs. Subgroup analysis showed that the EXOs derived from HMSCs, and that exosome therapy immediately after myocardial infarction can better improve the LVEF. Conclusions: EXOs therapy has the potential to improve cardiac function, fibrogenesis, and inflammatory response, as well as reducing cell apoptosis and autophagy in preclinical AMI animal models. This can inform future human clinical trials of EXOs.

**Supplementary Information:**

The online version contains supplementary material available at 10.1186/s13287-022-02833-z.

## Background

Although the best medical treatment and coronary artery reperfusion therapy improve the survival of patients, coronary heart disease is still one of the main causes of morbidity and mortality worldwide. Acute myocardial infarction (AMI) is characterized by the loss of cardiomyocytes, scarring, ventricular remodelling, and can develop into end-stage heart failure [1]. Therefore, effective cardiac repair is essential for the recovery of cardiac function after AMI. Many studies have confirmed that stem cell transplantation can improve cardiac tissue regeneration and cardiac function [2]. However, most of these studies have not found that stem cells have a direct role in heart colonization. Moreover, stem cell therapy may have certain risks; for example, it can induce arrhythmia and aggravate vascular blockage. Studies have shown that the improvement of heart function is not directly affected by transplanted stem cells. On the contrary, this improvement seems to be induced by paracrine factors, particularly exosomes (EXOs), secreted by stem cells [3].

Recently, literature data have emphasized that stem cell-derived exosomes can improve AMI in preclinical models. Exosomes are natural membranous nanoparticles [4], which are subgroups of extracellular vesicles encapsulated by lipid bimolecular membranes derived from endosomes, ranging in size from 30 to 150 nm. The contents of exosomes include lipids, proteins, mitochondrial DNA, microRNAs (miRNAs), mRNAs, and non-coding RNAs, which are important mediators in cardiac repair after AMI [5]. Moreover, exosomes are considered as new therapeutic candidates, playing an important role in intercellular and tissue-level communication in AMI stem cell therapy [6], while simultaneously overcoming some of the limitations of stem cell therapy.

Similar to human stem cell therapy, many researchers have studied the effectiveness of exosomes in AMI animal models [7]. Exosomes have been shown to participate in many cardiovascular physiological and pathological processes, including angiogenesis regulation, inflammation reduction, myocardial cell apoptosis inhibition, myocardial remodelling reduction, and the improvement of the microenvironment of infracted myocardium [8, 9]. These studies have addressed the unresolved issues of preclinical exosome therapy (i.e. cell type selection, exosome dosage, delivery method, treatment time, and follow-up after infarction). In order to provide the latest available evidence for clinical research, we conducted this meta-analysis to study the effectiveness of exosomes in preclinical animal models.

We performed a systematic overview of the pertinent literature, including a quantitative meta-analytical data analysis to assess the effects and the potential mechanisms of stem cell-derived exosome transplantation in AMI animal models. Our meta-analysis focused on the therapeutic effect of non-cardiac stem cell-derived exosomes on myocardial infarction. We believe that the meta-analysis of these preclinical data may be useful for designing future clinical studies.

## Methods

The systematic review protocol was registered in the International Prospective Register of Systematic Reviews (PROSPERO) website. This meta-analysis followed the Preferred Reporting Items for Systematic Reviews and Meta-Analyses criteria.

### Study selection

Two independent investigators (YZ and WW) conducted the study selection. Disagreements between investigators were resolved in meetings or adjudicated by a third reviewer (PC).

### Eligibility criteria

Inclusion criteria: (1) studies on preclinical AMI animal models; (2) studies on exosomes derived from stem cells (excluding cardiac stem cells); (3) studies providing detailed methods for the extraction and identification of exosomes; (4) studies containing the outcomes of the left ventricular ejection fraction (LVEF); (5) randomized controlled trials. Exclusion criteria: (1) studies on exosomes derived from cardiac stem cells, including cardiospheres, cardiac progenitor cells, cardiosphere-derived cells, etc.; (2) studies from which the data could not be extracted; (3) studies that are not controlled, such as case reports, reviews, meetings, letters, surveys, or satisfaction studies.

### Search strategy

We searched PubMed, EMBASE, and the Web of Science from database inception to September 1, 2021. The references of related reviews and meta-analyses were searched manually. The literature search strategy consisted of using the MeSH terms and the free words “myocardial infarction”, “extracellular vesicles” and “exosomes”, as shown in Additional file 1. The search was limited to references written in English. No ethical approval was required because the meta-analysis was based on published articles.

### Data abstraction

The following information was extracted from the complete manuscripts of the qualified studies: basal characteristics of the study, cardiac function, myocardial infarction, myocardial fibrosis, inflammatory expression, myocardial apoptosis, and autophagy. If necessary, data were estimated from graphics. For the final analysis, we used magnetic resonance imaging and echocardiography.

The following characteristics were analysed. For cardiac function, the LVEF and the fractional shortening (FS) were analysed. For myocardial infarction, the infarct size (IS) was analysed. The percentage of the IS was calculated as the sum of the infarct areas or area at risk from all sections/the sum of the LV areas from all sections × 100%. For myocardial fibrosis, the fibrosis area (FA) was analysed. The percentage of fibrotic area was calculated as the average ratio of the area of fibrosis or collagen to the area of the entire left ventricle. Regarding the inflammatory expression, the levels of TNF -α, IL-6 and IL-10 were analysed. For the apoptosis, the apoptosis rate was analysed. The apoptosis rate was quantified by the percentage of cells stained with TUNEL and cleaved caspase-3. For the autophagy flux, the number of autophagic vesicles was analysed.

### Quality assessment

The methodological quality of each included study was evaluated by three independent authors (FZ, YFZ and ML) using a Collaborative Approach to Meta-Analysis and Review of Animal Data from Experimental Studies (CAMARADES) 10-item checklist: A, peer-reviewed journal; B, temperature control; C, random allocation of animals; D, blind established models; E, blind outcome assessment; F, no obvious vascular protection when using anaesthetics; G, appropriate animal models (diabetes, advanced age, or hypertension); H, calculation of the sample size; I, statement of compliance with animal welfare regulations; and J, statement of potential conflicts of interest [10].

### Data analysis

Our main result is the difference in the mean LVEF of the animals in the control and treatment groups during follow-up. Secondary endpoints include the FS, IS, FA, the TNF-α, IL-6 and IL-10 levels, the apoptosis rate and the number of autophagic vesicles. We chose the study with the longest follow-up time. All results are continuous variables. The estimated effect size of continuous data is determined using the weighted mean difference (WMD) and the 95% confidence intervals (CIs). Unadjusted P-values are reported throughout, with hypothesis testing set at the two-tailed 0.05 level. Inconsistency was estimated by using the I^2^ statistic: values of 25%, 50% and 75% were considered low, moderate and high inconsistency, respectively, if heterogeneity was present (*I*^2^ > 50%) and random-effected models were used [11]. Subgroup and sensitivity analyses were performed to investigate the potential between-study heterogeneity and to explore other potentially confounding factors. A funnel plot was drawn for LVEF to explore the publication bias. The trim-and-fill computation method was used to estimate the effect of publication bias on the interpretation of the results.

All statistical analyses were conducted using Stata14.0 software (Stata Corporation, Texas). The data not provided in the studies were obtained by using Engauge Digitizer 10.8 and Origin 2017.

## Results

### Included study characteristics

**Fig. 1:**
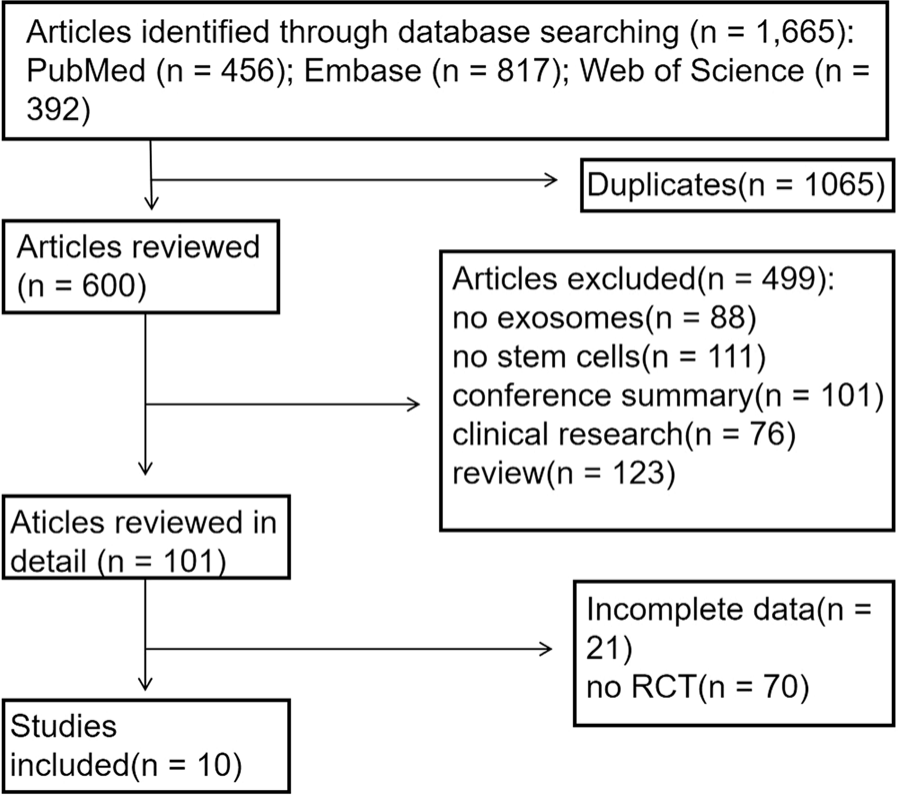
Flowchart of the enrolled studies on exosome therapy in animals with AMI. *RCT* randomized controlled trial

A total of 1665 articles were identified through the literature search, among which 10 articles were eligible for review, as shown in Fig. 1. A total of 167 animals were included in this meta-analysis. The main characteristics of the included studies are presented in Table 1. All exosomes were derived from stem cells. Four studies used human marrow mesenchymal stromal cells (HMSCs)-EXOs, two studies used animal mesenchymal stromal cells (AMSCs)-EXOs, one study used adipose-derived stem cells (ADSCs)-EXOs and two studies used engineered mesenchymal stem cells (EMSCs)-EXOs. All studies explained the methods of extraction and identification of exosomes in detail. The morphology of exosomes was observed using transmission electron microscope, the size and quantity of exosomes were measured using Exosome Nanoparticle Tracking Analysis (NTA), and the surface antigens of exosomes were detected using western blots. Nine studies used ultracentrifugation, and one study used an exosome isolation reagent. The diameter of the isolated EXOs mostly ranged from 30 to 150 nm. Surface markers, including CD9, CD63, CD81, Alix-101, and TSG-101, were used to identify and sort the EXOs from other components. Two studies were divided into normal oxygen-induced exosome group (N-exo group) and hypoxia-induced exosome group (H-exo group). We only analysed the N-exo group. All studies reported the establishment of an AMI animal model by performing thoracotomy and the ligation of the left descending coronary artery, for which nine studies reported using the permanent left anterior descending (LAD) ligation model and one reported using the ischemia–reperfusion model. About the administration methods of EXOs, eight studies used instant intracardiac injection in the border zone after LAD ligation, and two used intravenous injection via the tail vein. There were large differences in the total doses of EXOs, varying from 0.002 to 2 µg/µL. The LVEF was reported in ten studies and the FS was reported in nine studies. The timing of cell therapy after the induction of MI was one of the following: immediately (3 studies), 30 min (3 studies), or 60 min (4 studies). The median follow-up imaging time was 3 weeks (range 2–4 weeks).

**Table 1:** Characteristics of included studies

Study	Region	Type	Model	Species sex	Source	Exosome marker	Dose	Route	Therapy time (min)	Follow-up (weeks)
Xiao [12]	China	LAD	MI	male mice	EMSCs	miR-125b	0.2 μg/μL	IM	30	4
Liu [10]	Netherlands	LCX	MI	pig	HMSCs	N	0.002 μg/μL	IV	60	1, 4
Zhang [17]	China	LAD	MI	male rat	EMSC	miR-24	NSI	IM	60	1, 4
Huang [15]	China	LAD	MI	male mice	ADSCs	N	5 μg/μL	IM	60	4
Sun [18]	China	LAD	MI	male rat	HMSC	miR-873	1 μg/μL	IM	0	4
Huang [21]	China	LAD	MI	female rat	AMSC	lncRNA H19	0.1 μg/μL	IM	30	4
Liu [20]	China	LAD	MI	rat	HMSC	N	1 μg/μL	IM	0	4
Wang [13]	China	LAD	MI	male rat	HMSC	miR-125b	2 μg/μL	IV	0	4
Li [16]	China	LAD	MI	male rat	AMSC	miR-301	NSI	IM	30	4
Wei [19]	China	LAD	I/R	male rat	HMSC	miR-181a	NSI	IM	45	1

*IM* myocardial injection, *IV* intravenous injection

### Quality of the included studies

All the included records were peer-reviewed publications, and all animals were allocated randomly to treatment and control groups. The details of the study quality assessment are shown in Additional file 2. The average impact factor of the journals was 13.47.

### Meta-analyses

#### Outcome

**Fig. 2:**
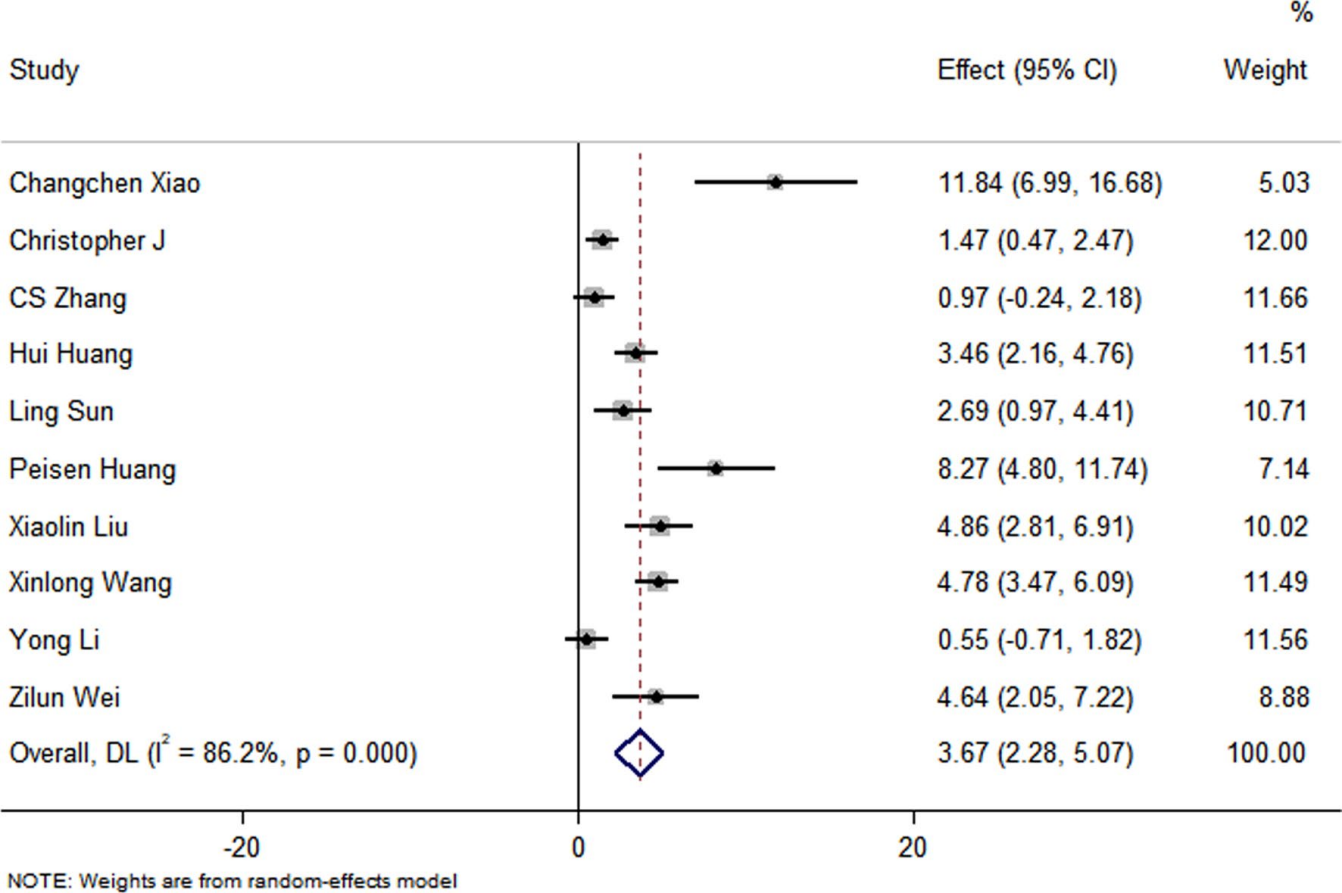
Forest plot showing the impact of exosome therapy on LVEF improvement, compared with controls. WMD = 3.67%; 95% CI 2.28–5.07%; test of overall effect, *P* = 0.000; *I*^2^ = 86.2%, *P* = 0.000. 95% CI 95% confidence interval

**Fig. 3:**
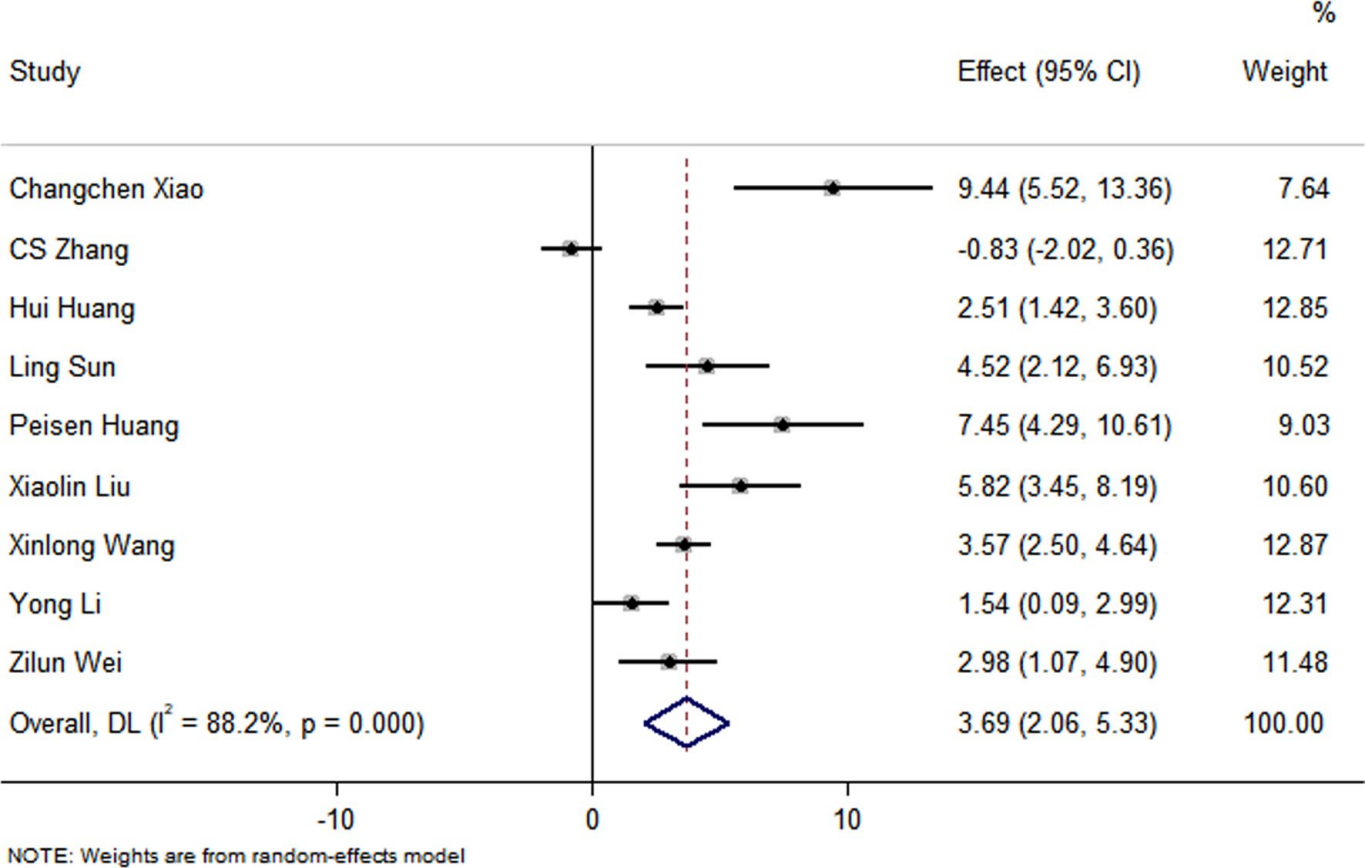
Forest plot showing the impact of exosome therapy on FS improvement, compared with controls. WMD = 3.69%; 95% CI 2.06–5.33%; Test of overall effect, *P* = 0.000; *I*^2^ = 88.2%, *P* = 0.000

**Fig. 4:**
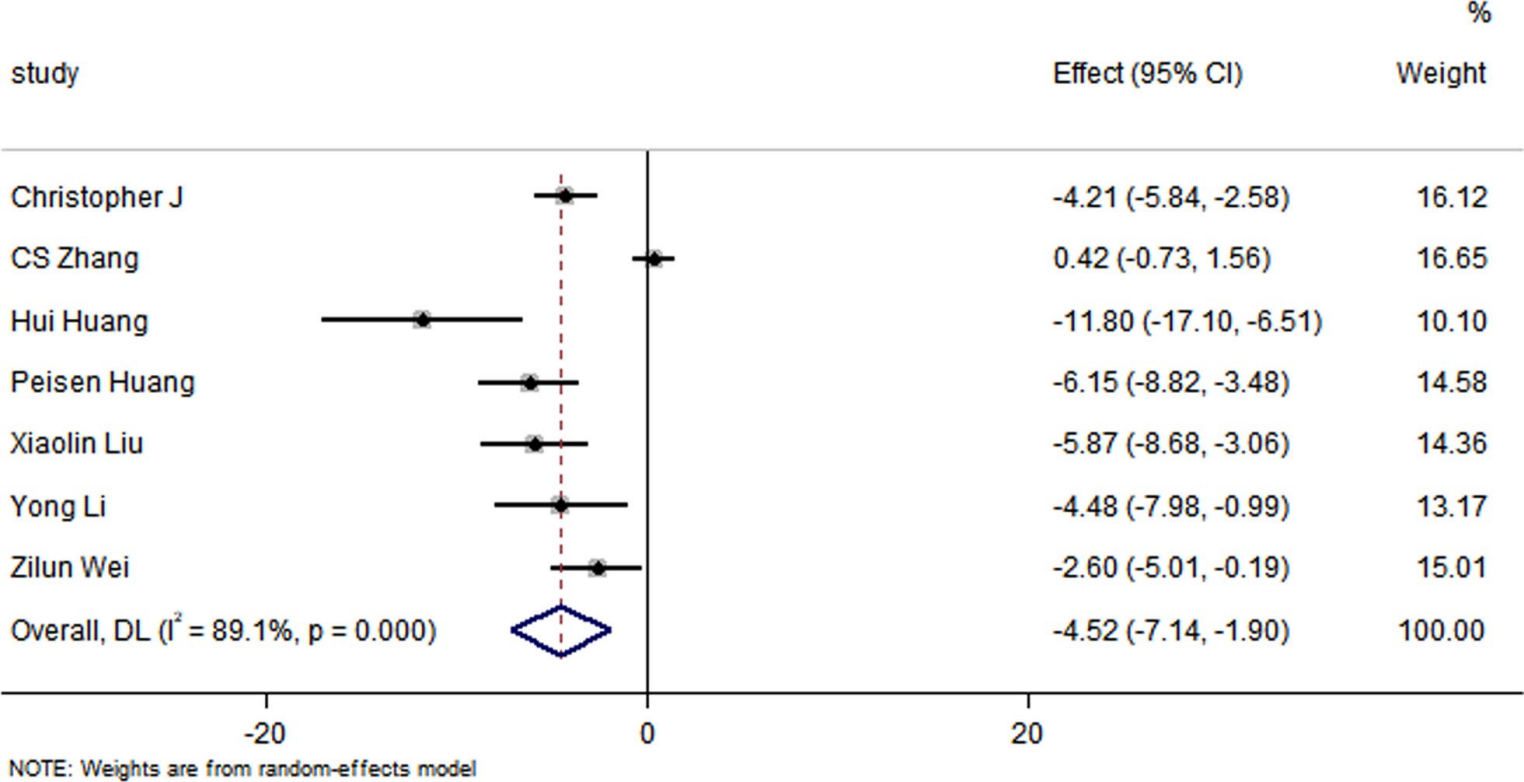
Forest plot showing the impact of exosome therapy on IS improvement, compared with controls. WMD = −4.52%, 95% CI − 7.14 to − 1.9%; Test of overall effect, *P* = 0.001; *I*^2^ = 89.1%, *P* = 0.000

**Fig. 5:**
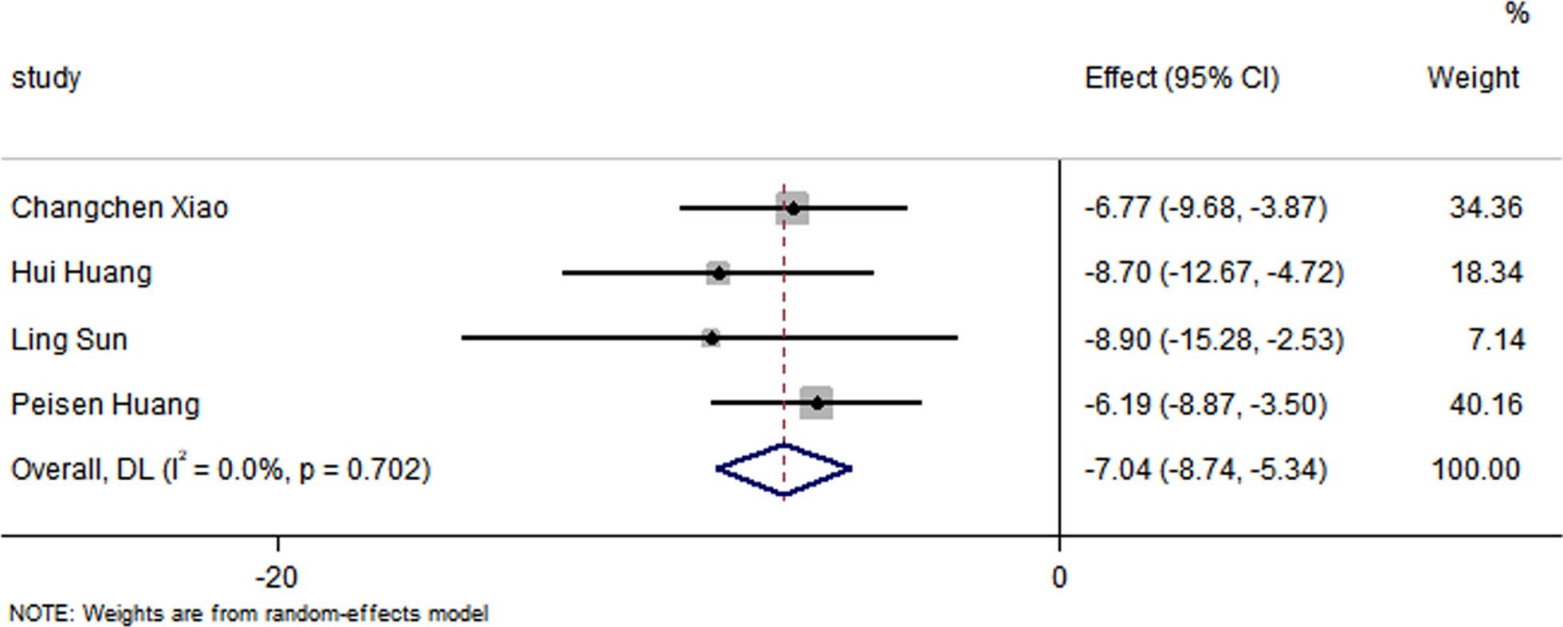
Forest plot showing the impact of exosome therapy on the FA improvement, compared with controls. WMD = −7.04%, 95% CI − 8.74 to − 5.34%; Test of overall effect, *P* = 0.000; *I*^2^ = 0.0%, *P* = 0.702

**Fig. 6:**
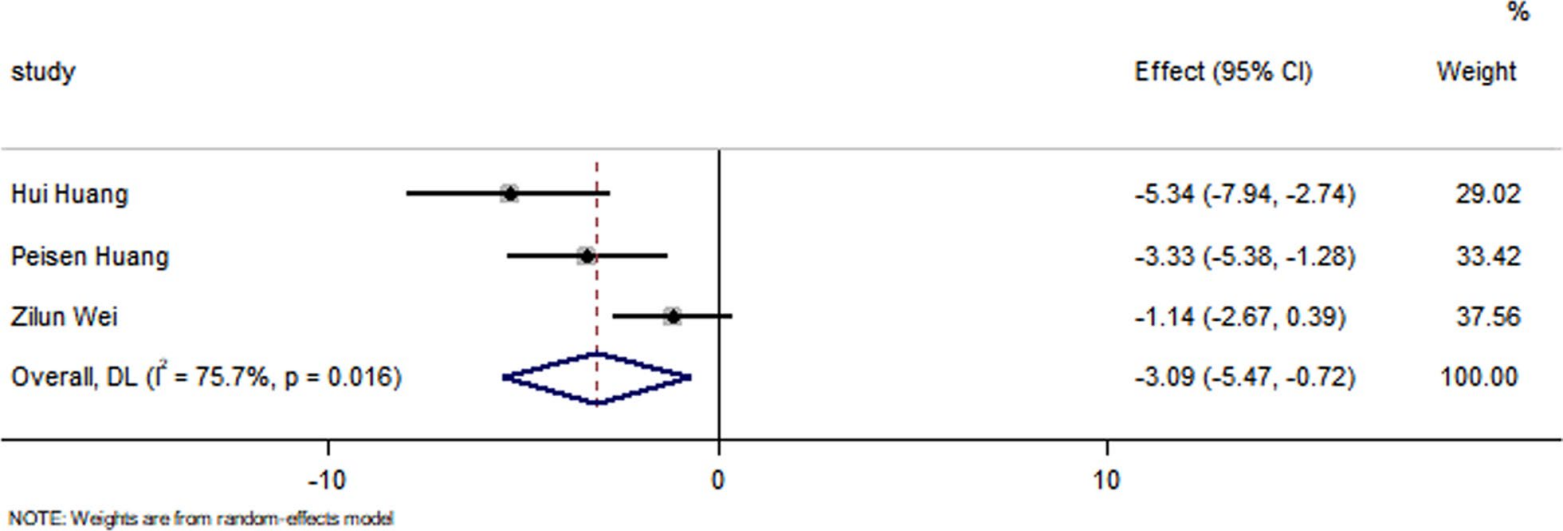
Forest plot showing that exosome therapy reduced the level of TNF-α compared with controls. WMD = −3.09, 95% CI − 5.47 to − 0.72; Test of overall effect, *P* = 0.011; *I*^2^ = 75.7%, *P* = 0.016

**Fig. 7:**
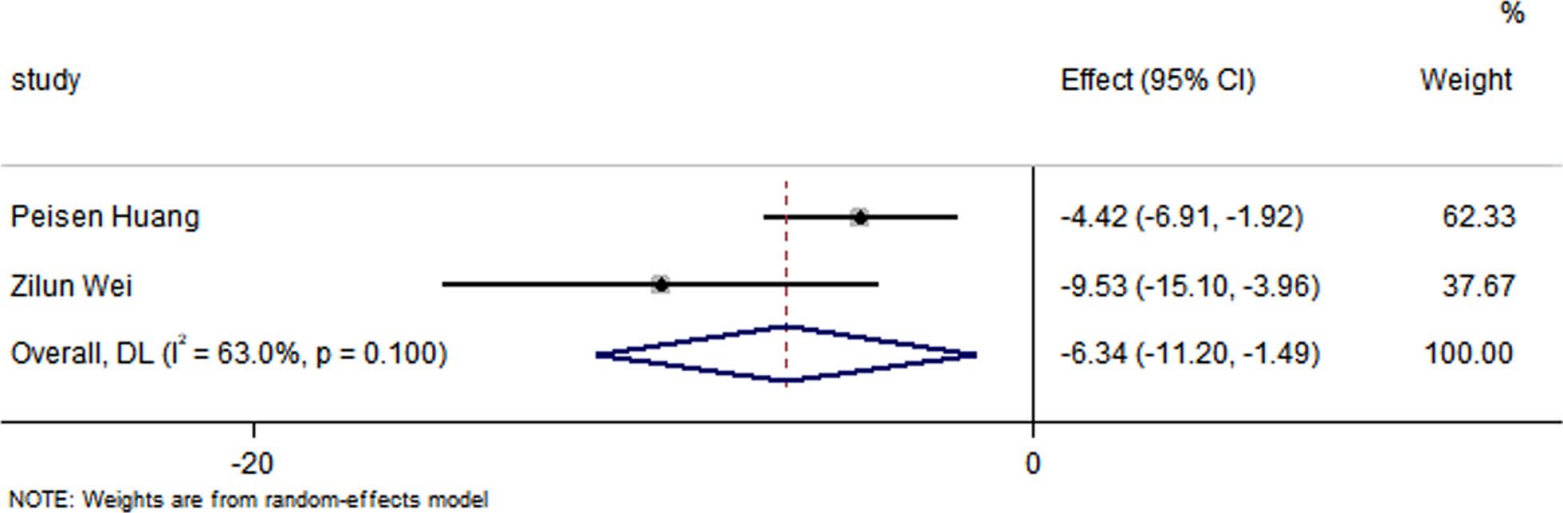
Forest plot showing that exosome therapy reduced the level of IL-6 compared with controls. WMD = −6.34, 95% CI − 11.2 to − 1.49; Test of overall effect, *P* < 0.01; *I*^2^ = 63%, *P* = 0.1

**Fig. 8:**
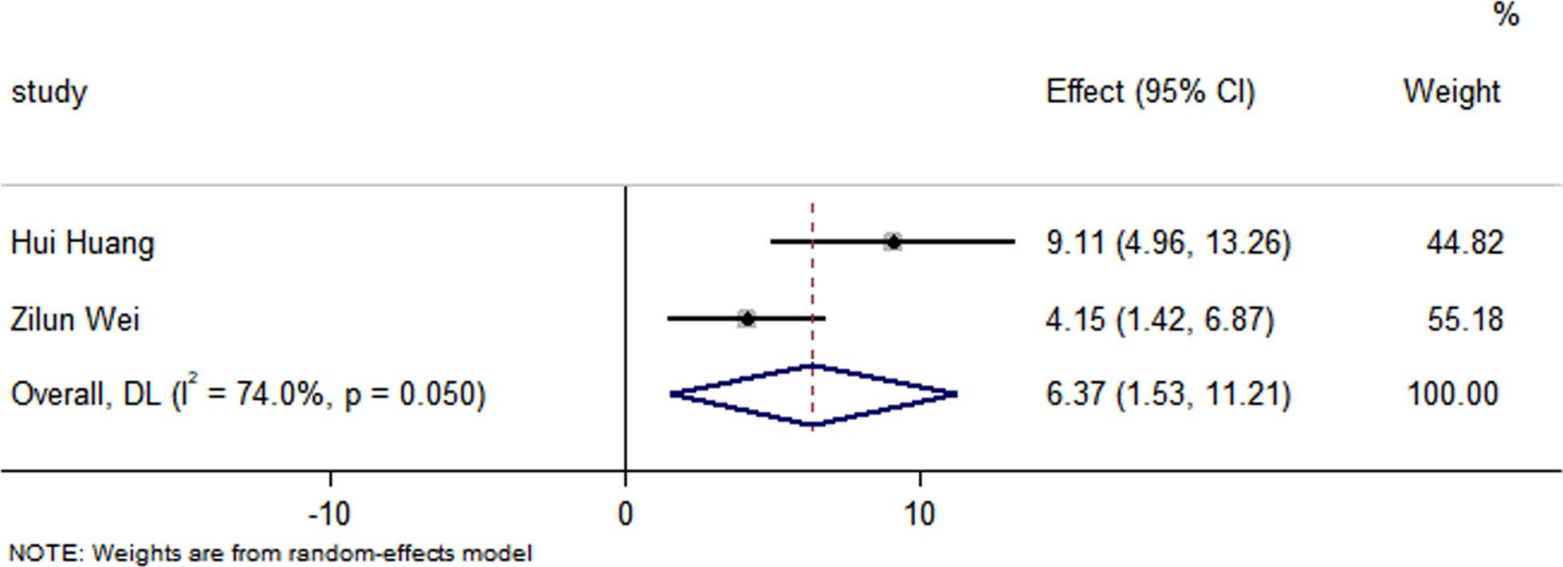
Forest plot showing that exosome therapy increased the level of IL-10 compared with controls. WMD = 6.37, 95% CI 1.53–11.21;Test of overall effect, *P* = 0.01; *I*^2^ = 74%, *P* = 0.05

**Fig. 9:**
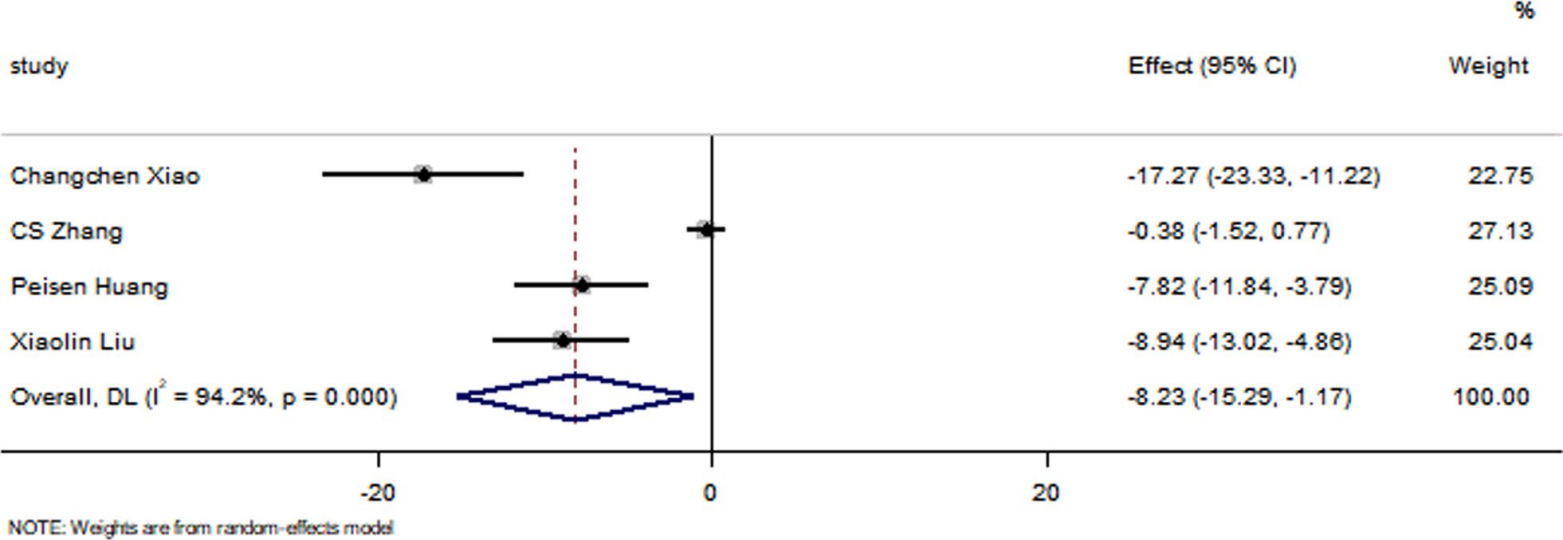
Forest plot showing that exosome therapy improved apoptosis compared with controls. WMD = −8.23, 95% CI − 15.29 to − 1.17; Test of overall effect, *P* = 0.000; *I*^2^ = 94.2%, *P* = 0.000

**Fig. 10:**
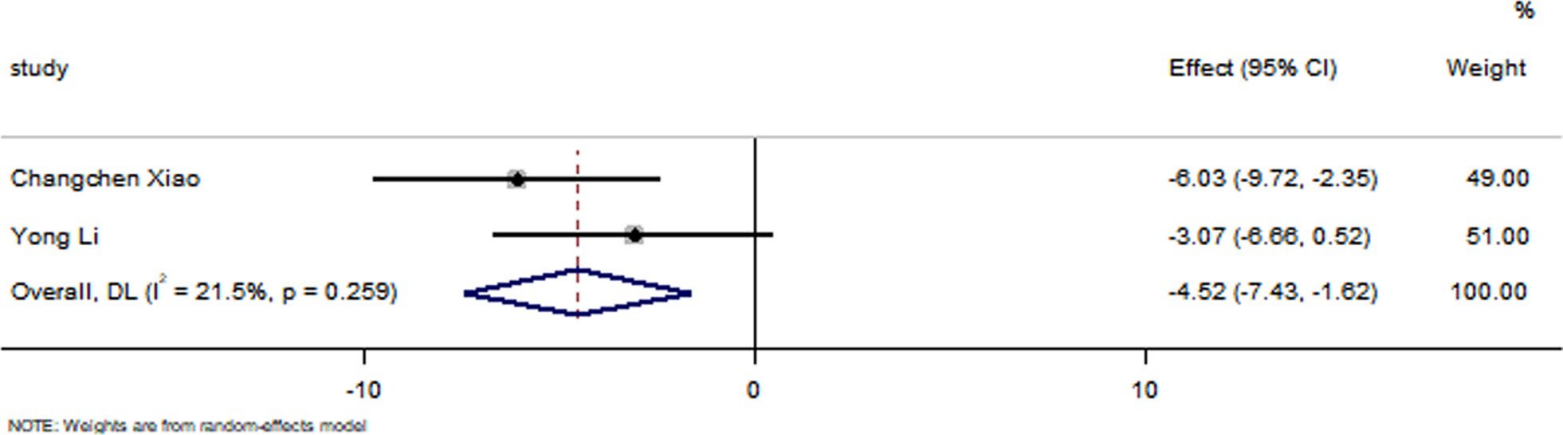
Forest plot showing that exosome therapy improved autophagy compared with controls. WMD = −4.52, 95% CI − 7.43 to − 1.62; Test of overall effect, *P* = 0.000; *I*^2^ = 21.5%, *P* = 0.259

All studies have reported no significant differences in the LVEF at baseline between the control group and the EXO-treated group (*P* > 0.05). Pooled analysis showed a LVEF difference of 3.67% at follow-up after exosome therapy compared with the control group value (95% CI 2.28–5.07%; *P* = 0.000) with significant heterogeneity (*P* = 0.000) and inconsistency (*I*^2^ = 86.2%). An FS difference of 3.69% was verified at follow-up after exosome therapy compared with the control group value (95% CI 2.06–5.33%; *P* = 0.000) with significant heterogeneity (*P* = 0.000) and inconsistency (*I*^2^ = 88.2%). The IS was significantly decreased in the exosome therapy group (WMD = −4.52%, 95% CI − 7.14 to − 1.9%; *P* = 0.001). The FA was significantly decreased in the exosome therapy group (WMD = −7.04%, 95% CI − 8.74 to − 5.34%; *P* = 0.000). The level of TNF-α was significantly decreased in the exosome therapy group (WMD = −3.09, 95% CI − 5.47 to − 0.72; P = 0.011). The level of IL-6 was significantly decreased in the exosome therapy group (WMD = −6.34, 95% CI − 11.2 to − 1.49; *P* < 0.01). The level of IL-10 was significantly increased in the exosome therapy group (WMD = 6.37, 95% CI 1.53–11.21; *P* = 0.01). The apoptosis rate was significantly decreased in the exosome therapy group (WMD = −8.23, 95% CI − 15.29 to − 1.17; *P* = 0.000). The number of autophagic vesicles was significantly decreased in the exosome therapy group (WMD = −4.52, 95% CI − 7.43 to − 1.62; *P* < 0.01), as shown in Figs. 2, 3, 4, 5, 6, 7, 8, 9 and 10.

#### Sensitivity analyses

**Fig. 11:**
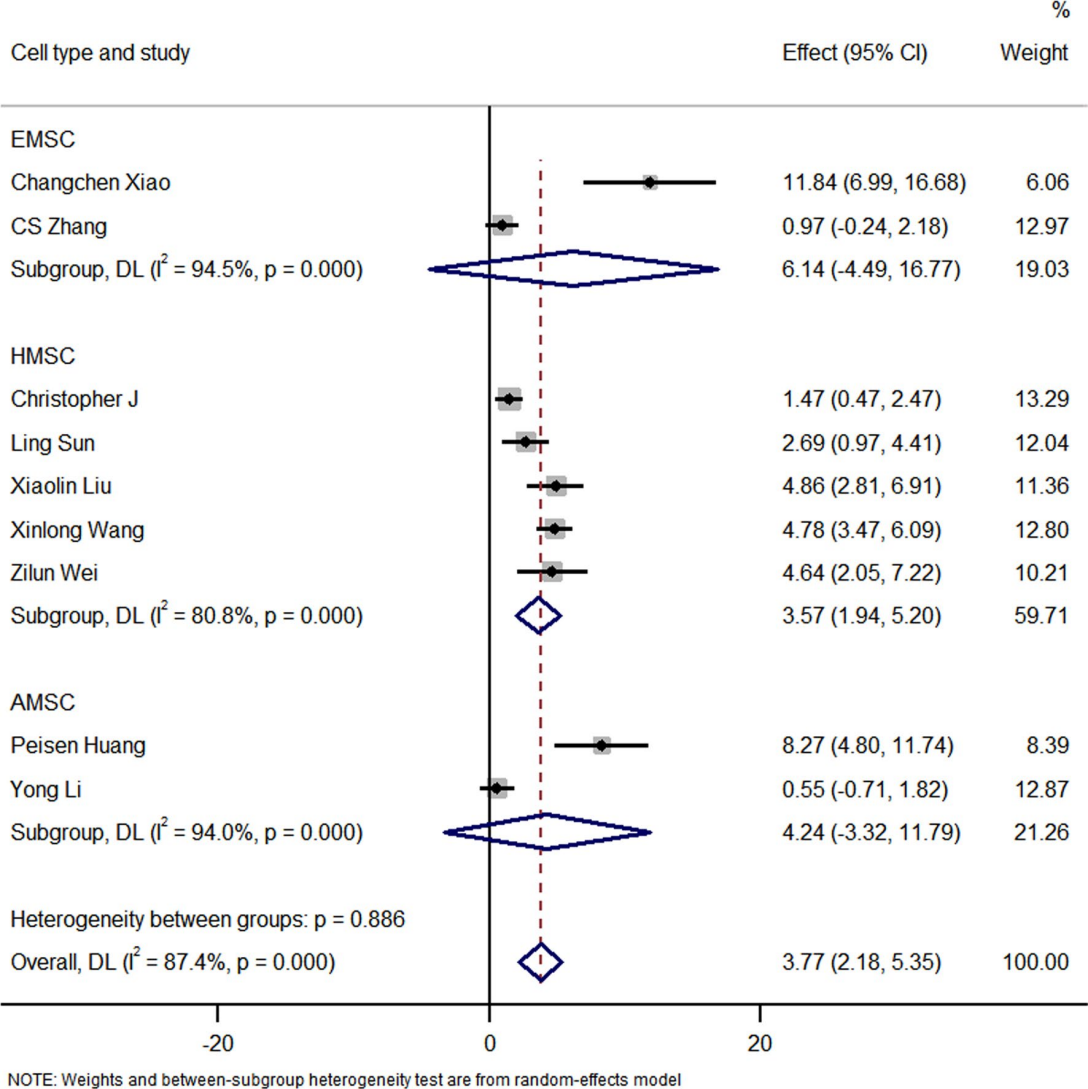
Subgroup analysis showing the effect of cell type. Compared with the control group, exosome therapy improved LVEF (*P* = 0.000). The exosome therapy derived from HMSC significantly improved LVEF compared with the controls (*P* = 0.000). There was no significant difference between the treatment of exosomes derived from AMSC (*P* = 0.258) or EMSC (*P* = 0.272) and the control group

**Fig. 12:**
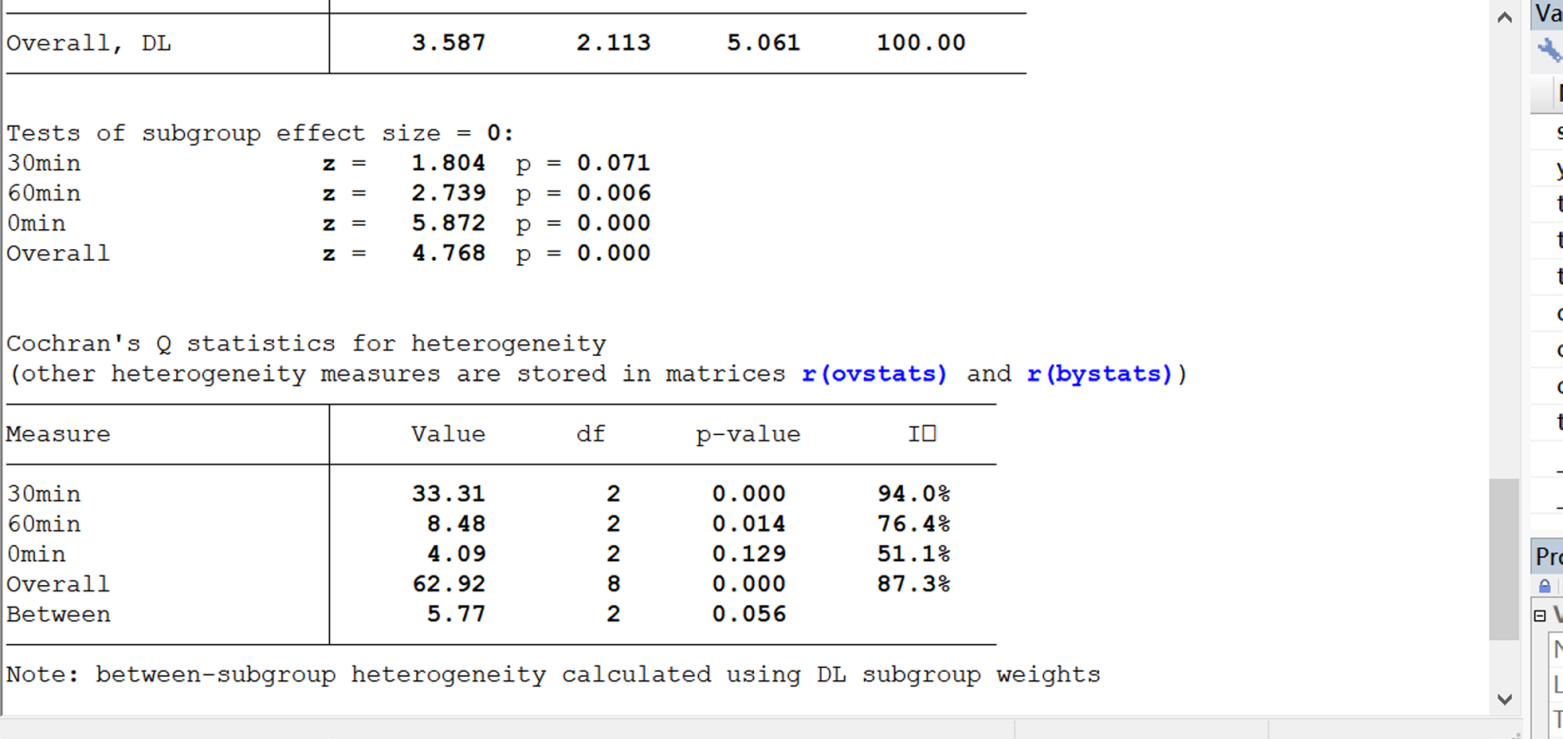
Subgroup analysis showing the effects of therapy timing. Compared with the control group, exosome therapy at 0 (*P* = 0.000) and 60 (*P* = 0.006) mins after myocardial infarction significantly improved LVEF. Exosome therapy 30 min (*P* = 0.071) after myocardial infarction did not significantly improve LVEF

**Fig. 13:**
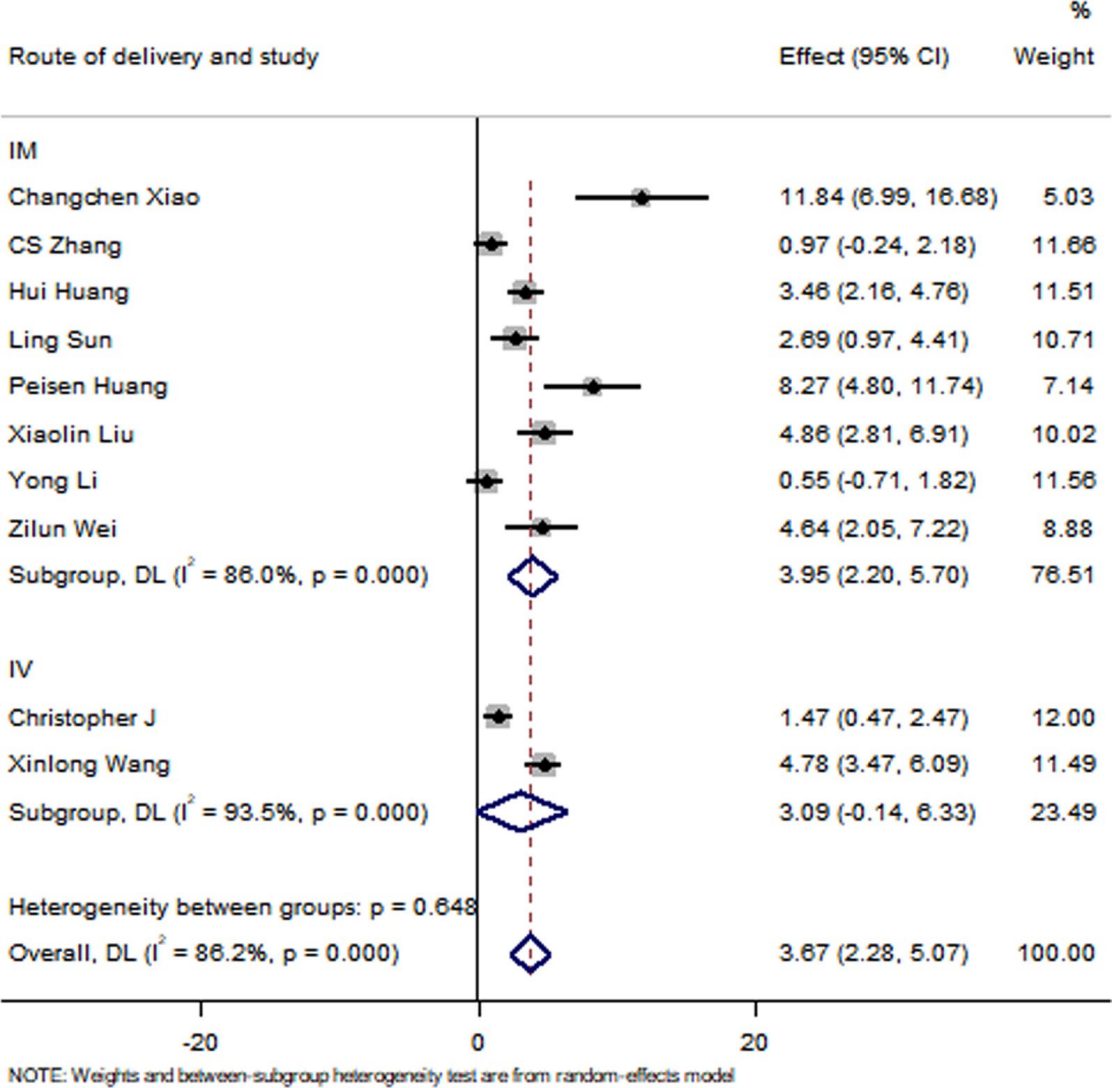
Subgroup analysis showing the effects of the injection mode. After intramuscular injection of exosomes in patients with myocardial infarction, the improvement of LVEF in the exosome treatment group was significantly higher than in the control group (*P* = 0.000). Compared with the control group, the LVEF of the exosome-treatment group was not significantly improved after intravenous injection of exosomes (*P* = 0.061)

**Fig. 14:**
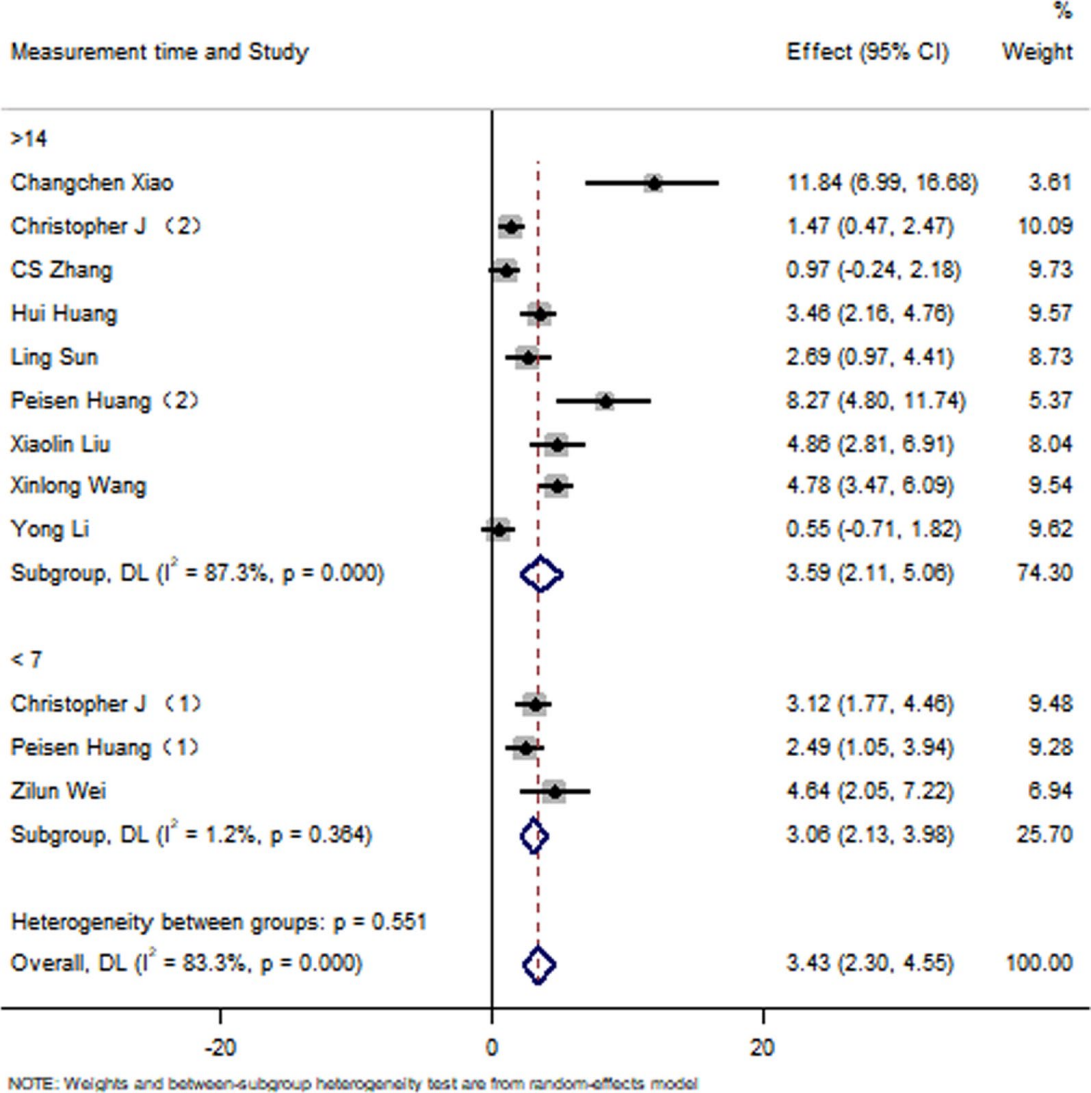
Subgroup analysis showing the effect of the follow-up duration. Compared with the control group, LVEF in the exosome-treatment group was significantly improved, whether followed up for < 7 days (*P* = 0.000) or for > 14 days (*P* = 0.000)

**Fig. 15:**
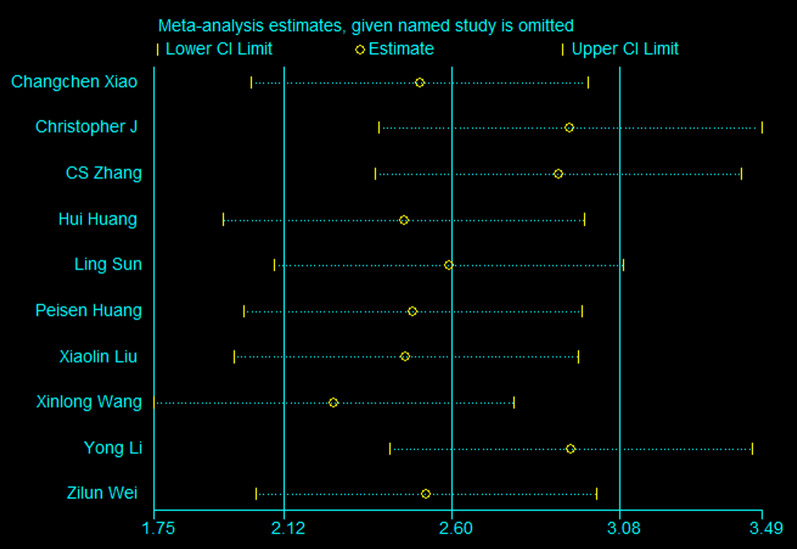
Sensitivity-analysis results. The sensitivity analysis showed that no single study significantly influenced the results

**Fig. 16:**
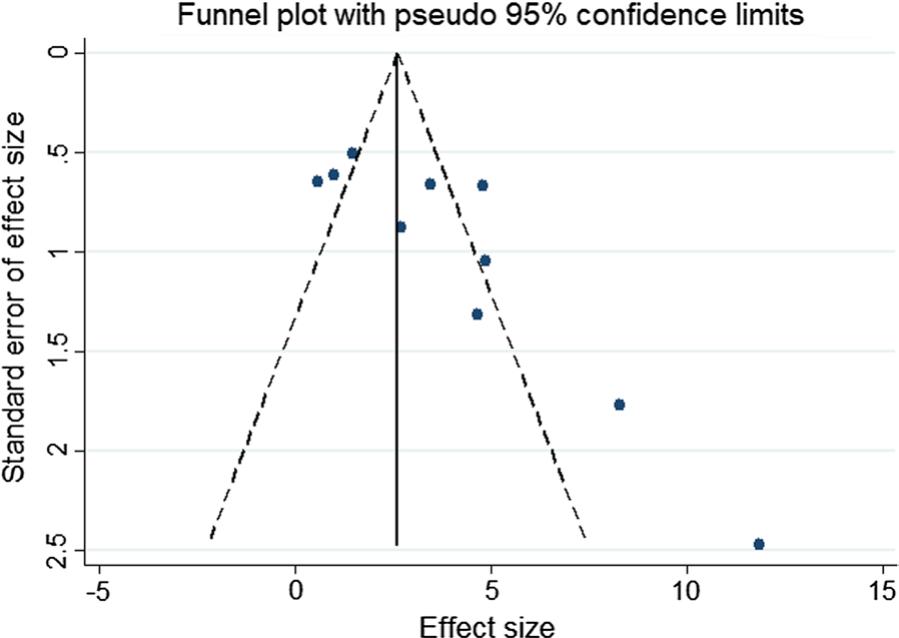
Publication-bias analysis results. The black spots in the figure are not evenly distributed on both sides of the funnel, suggesting publication bias

The subgroup analysis showed that the EXOs derived from HMSCs can significantly improve the LVEF. Moreover, exosome therapy immediately after myocardial infarction can better improve the LVEF. However, there were no differences between the exosome injection method results of the two groups. Interestingly, a follow-up time of < 7 days and > 14 days in the exosome therapy group resulted in an improved LVEF compared with that of the control group. None of the studies presented a follow-up of 7–14 days. The sensitivity analysis showed that none of the single studies significantly influenced the results. The funnel plot for LVEF suggests that there is publication bias in this analysis, as shown in Figs. 11, 12, 13, 14, 15 and 16. However, further analysis using the trim-and-fill method indicated that it did not impact the estimates (i.e. no trimming was done because the statistical results were unchanged), as shown in Additional file 3.

## Discussion

This meta-analysis article comprises data from 10 published preclinical randomized controlled trial (RCT) studies [12–21]. The main research findings are as follows: (1) exosome transplantation can make the LVEF and FS increase by 3.67% and 3.69%, respectively; (2) exosome transplantation can reduce the infarct size by 4.52%; (3) exosome transplantation is involved in the regulation of pathophysiological processes after myocardial infarction, including the inflammatory response, apoptosis and autophagy; (4) sensitivity analysis suggests that the cell type and treatment time have beneficial effects on the LVEF; (5) while the follow-up time and delivery route do not.

### The efficacy and potential mechanisms of exosome therapy in preclinical trials

The overall beneficial effect of exosome therapy has been observed in this analysis. Exosome therapy can improve cardiac function, which manifests as an improvement in the LVEF and FS. Further research found that the area of myocardial infarction in the exosome treatment group was smaller than that in the control group, and that the exosome treatment group had less fibrous proliferation.

These results suggest that exosomes may inhibit myocardial cell death and promote myocardial repair to a certain extent. Apoptosis occurs after myocardial ischemia, and an imbalance of apoptosis will promote myocardial infarction. The infracted myocardium stimulates the proliferation of collagen fibres and promotes changes in the cardiac outcome. Studies have confirmed that miR-126, mir-133a and miR-499 in exosomes are involved in the process of inhibiting apoptosis and fibre proliferation [22].

The levels of IL-6 and TNF-α in the exosome transplantation group were lower than those in the control group, indicating that exosomes may be involved in regulating the inflammatory response. Interestingly, the level of IL-10, an inflammatory inhibitor, of the exosome treatment group was higher than that of the control group. After coronary artery ligation, vascular endothelial cells undergo structural dysfunction due to ischemia and hypoxia, thereby activating the immune system. Through the crosstalk between cardiomyocytes, the release of various pro-inflammatory mediators increases. Cardiomyocytes release chemokines and bind to related chemokine receptors to up-regulate the expression of pro-inflammatory factors (such as TNF, IL-1B and IL-6), allowing a large number of inflammatory cells to accumulate in the infracted myocardium. Exosomes may improve cardiac function after myocardial infarction by regulating the expression of inflammatory factors.

Autophagy is a physiological process that produces ATP and induces protein synthesis by degrading the circulating cytoplasmic proteins and damaged organelles. Autophagy can increase protein degradation, reduce myocardial hypertrophy, and antagonize ventricular hypertrophy. Low autophagy levels can reduce the damage of cardiomyocytes under pressure overload, while excessive autophagy levels make the heart more vulnerable to injury and dysfunction. Under severe ischemia and hypoxia conditions, excessive autophagy promotes the absorption of dead cells by macrophages, activates the inflammatory response, leads to system disorders, and promotes and aggravates heart failure. In AMI animal model studies, the autophagy level of the exosome treatment group was lower than that of the control group. Exosomes may improve excessive autophagy under ischemia and hypoxia conditions.

Hypoxia after myocardial infarction can stimulate the expression of growth factors, promote the proliferation of endothelial cells and smooth muscle cells, and promote angiogenesis. Stem cell exosomes seem to stimulate angiogenesis in the area around the infarct area. The expression of specific cytokines representative of vascular proliferation was observed in the exosome transplantation group. Some studies also believe that the exosomes obtained from human umbilical cord mesenchymal stem cells can activate platelet-derived growth factor D to enhance heart regeneration and promote angiogenesis [23]. Various vasoactive factors have been found in exosomes. Promoting angiogenesis may be a common and critical function of stem cell-derived exosomes.

In the past, the stem cell treatment of AMI was thought to replenish new cardiomyocytes through differentiation, thereby promoting heart repair. However, recent studies have shown that stem cells achieve their cardioprotective effect mainly by secreting paracrine factors such as exosomes. In recent years, stem cell exosomes have been shown to reduce the area of myocardial infarction and improve cardiac function after myocardial infarction. This indicates that the exosome-based method has broad prospects as a potential new cellular therapy for heart repair.

**Fig. 17:**
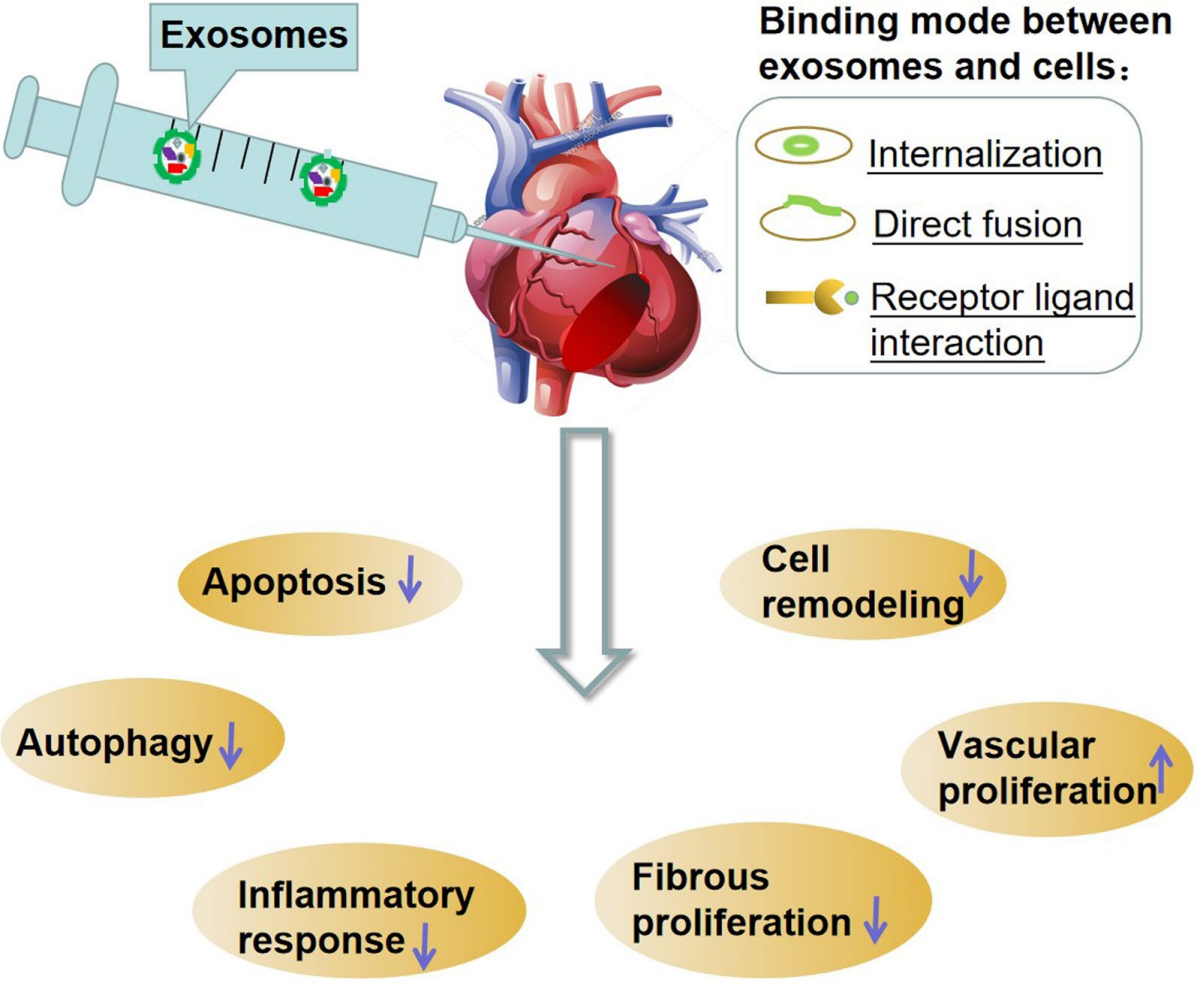
Possible mechanism of exosome treatment of AMI

Therefore, we know that exosomes are involved in the regulation of pathophysiological processes after AMI, including apoptosis, fibre proliferation, inflammatory response, vascular proliferation and autophagy (Fig. 17).

### Recommendations for future research on translational exosomes

Exosomes must undergo preclinical research before they are used in clinical studies. We believe that in preclinical studies, the following criteria should be used for exosome studies: a randomized study design should be adopted; blind functional analysis should be performed; the timing of exosome injection, source and identification methods of exosomes should be reported; the adverse events and mortality should be recorded; and different follow-up times should be used. In future studies, large animal studies and clinical trials should focus on the human MSC-derived exosome transplantation, as well as on the later injection time points (within 30 min after AMI). The follow-up time should be set at less than 7 days, 7 to 14 days, and more than 14 days. The main indicators of the evaluation experiment include the ejection fraction, the myocardial infarction area, and the secondary indicators include the level of inflammatory factors, the rate of apoptosis or apoptotic proteins, the number of autophagosomes and the blood vessel density.

Meta-analyses of animal studies can often guide research and clinical endeavours. Performing a preclinical meta-analysis also evaluates the safety of exosome therapy in order to design future clinical trials.

### Limitations

The limitations of meta-analyses are well known. In our research, in particular, different methods and materials, such as the stem cell types, administration methods, injection time after myocardial infarction, follow-up time after cell therapy, exosome extraction methods and detection tools, have been used. All of these have an impact on the observation results of this study. Our research is highly heterogeneous. Through random effects analysis, it was found that the risk of incorrect estimation is minimal. We also found some sources of heterogeneity (including the cell type and treatment time) using a variety of methods. In our study, fewer studies and animals were included. Most of the animals we studied were mice. Therefore, our results have some limitations. RCT studies of large animals using a large number of animals are urgently needed. Our research has publication bias; however, the shear complementation method results show that our results are stable. Studies have shown that the injection of exosomes of different species can cause an immune response; nonetheless, in our research, we showed that the injection of human mesenchymal stem cell-mediated exosomes can improve the cardiac function of mice. This may be because the study we included did not take into account the complications and mortality resulting from exosome treatment. We hope that further studies are done in the future to supplement our results. Finally, the follow-up time of animal studies is relatively short. Due to the shortcomings of this meta-analysis—including the wide span in injected exosome dosages, the small number of included studies, and the high heterogeneity—the analysis results should be interpreted with care. We trust that these deficiencies can be improved in future research. This analysis suggests that stem cell–derived exosome therapy may improve the LVEF, and it provides important clues for the design of (pre-) clinical trials.

## Conclusions

To the best of our knowledge, this is the first systematic review and meta-analysis of the effects of exosomes in the treatment of AMI in animal models. This analysis suggests that stem cell–derived exosome therapy may improve the LVEF, and it provides important clues for the design of (pre-) clinical trials.

## Supplementary Information

**Additional file 1.** Search strategy.

**Additional file 2.** Quality of eligible studies.

**Additional file 3.** Trim-and-fill test.

## Data Availability

The authors report no conflicts of interest. The authors alone are responsible for the content and the writing of the paper.

## Abbreviations

AMI: Acute myocardial infarction
CI: Confidence intervals
FA: Fibrosis area
FS: Fractional shortening
IS: Infarct size
LVEF: Left ventricular ejection fraction
WMD: Weighted mean difference
AMSC: Animal mesenchymal stromal cells
HMSC: Human marrow mesenchymal stromal cells
LAD: Left anterior descending
RCT: Randomized controlled trial
LCX: Left circumflex branch
I/R: Ischemia reperfusion;
EMSC: Engineered mesenchymal stem cells

## References

[1] Zheng YL, Wang WD, Li MM, Lin S, Lin HL (2021). Updated role of neuropeptide Y in nicotine-induced endothelial dysfunction and atherosclerosis. Front Cardiovasc Med.

[2] Lalu MM, Mazzarello S, Zlepnig J, Dong YY, Montroy J, McIntyre L, Devereaux PJ, Stewart DJ, David Mazer C, Barron CC, McIsaac DI, Fergusson DA (2018). Safety and efficacy of adult stem cell therapy for acute myocardial infarction and ischemic heart failure (SafeCell Heart): a systematic review and meta-analysis. Stem Cells Transl Med.

[3] Sahoo S, Losordo DW (2014). Exosomes and cardiac repair after myocardial infarction. Circ Res.

[4] Isaac R, Reis FCG, Ying W, Olefsky JM (2021). Exosomes as mediators of intercellular crosstalk in metabolism. Cell Metab.

[5] Guo D, Xu YR, Ding J, Dong JY, Jia N, Li Y, Zhang MM (2020). Roles and clinical applications of exosomes in cardiovascular disease. Biomed Res Int.

[6] Pan W, Zhu YJ, Meng XM, Zhang CL, Yang Y, Bei YH (2019). Immunomodulation by exosomes in myocardial infarction. J Cardiovasc Transl Res.

[7] Xiong YY, Gong ZT, Tang RJ, Yang YJ (2021). The pivotal roles of exosomes derived from endogenous immune cells and exogenous stem cells in myocardial repair after acute myocardial infarction. Theranostics.

[8] Gao YJ, Wu DD, Jia DD, Guo QQ, Wang MM, Yang R, Zhang XY, Chen M, Zhang DM (2021). Hypoxic stem cell-derived extracellular vesicles for cardiac repair in preclinical animal models of myocardial infarction: a meta-analysis. Stem Cells And Dev.

[9] Zhu LP, Tian T, Wang JY, He JN, Chen T, Pan M, Xu L, Zhang HX, Qiu XT, Li CC, Wang KK, Shen H, Zhang GG, Bai YP (2018). Hypoxia-elicited mesenchymal stem cell-derived exosomes facilitates cardiac repair through miR-125b-mediated prevention of cell death in myocardial infarction. Theranostics.

[10] Liu C, Wang J, Hu J, Fu B, Mao Z, Zhang HD, Cai GY, Chen XM, Sun XF (2020). Extracellular vesicles for acute kidney injury in preclinical rodent models: a meta-analysis. Stem Cell Res Ther.

[11] Zgliczynska M, Zgliczynski S, Ciebiera M, Kosinska-Kaczynska K (2019). Occupational burnout syndrome in polish physicians: a systematic review. Int J Environ Res Public Health.

[12] Xiao CC, Wang K, Xu YC, Hu HX, Zhang N, Wang YC, Zhong ZW, Zhao J, Li QJ, Zhu D, Ke CL, Zhong SH, Wu XP, Yu H, Zhu W, Chen JH, Zhang JY, Wang JA, Hu XY (2018). Transplanted mesenchymal stem cells reduce autophagic flux in infarcted hearts via the exosomal transfer of miR-125b. Circ Res.

[13] Wang XL, Zhao YY, Sun L, Shi Y, Li ZQ, Zhao XD, Xu CG, Ji HG, Wang M, Xu WR, Zhu W (2018). Exosomes derived from human umbilical cord mesenchymal stem cells improve myocardial repair via upregulation of Smad7. Int J Mol Med.

[14] Charles CJ, Li RR, Yeung T, Mazlan SMI, Lai RC, de Kleijn DPV, Lim SK, Richards AM (2020). Systemic mesenchymal stem cell-derived exosomes reduce myocardial infarct size: characterization with MRI in a porcine model. Front Cardiovasc Med.

[15] Huang H, Xu ZX, Qi Y, Zhang W, Zhang CJ, Jiang M, Deng SQ, Wang HR (2020). Exosomes from SIRT1-overexpressing ADSCs restore cardiac function by improving angiogenic function of EPCs. Mol Therapy Nucleic Acids.

[16] Li Y, Yang R, Guo BY, Zhang HB, Zhang H, Liu SY, Li YJ (2019). Exosomal miR-301 derived from mesenchymal stem cells protects myocardial infarction by inhibiting myocardial autophagy. Biochem Biophys Res Commun.

[17] Zhang CS, Shao K, Liu CW, Li CJ, Yu BT (2019). Hypoxic preconditioning BMSCs-exosomes inhibit cardiomyocyte apoptosis after acute myocardial infarction by upregulating microRNA-24. Eur Rev Med Pharmacol Sci.

[18] Sun L, Zhu W, Zhao P, Wang Q, Fan B, Zhu Y, Lu Y, Chen Q, Zhang J, Zhang F (2020). Long noncoding RNA UCA1 from hypoxia-conditioned hMSC-derived exosomes: a novel molecular target for cardioprotection through miR-873-5p/XIAP axis. Cell Death Dis.

[19] Wei ZL, Qiao SH, Zhao JX, Liu YH, Li QL, Wei ZH, Dai Q, Kang LN, Xu B (2019). miRNA-181a over-expression in mesenchymal stem cell-derived exosomes influenced inflammatory response after myocardial ischemia-reperfusion injury. Life Sci.

[20] Liu XL, Li X, Zhu WW, Zhang YL, Hong YM, Liang XT, Fan BH, Zhao HY, He HW, Zhang FX (2020). Exosomes from mesenchymal stem cells overexpressing MIF enhance myocardial repair. J Cell Physiol.

[21] Huang PS, Wang L, Li Q, Tian XQ, Xu J, Xu JY, Xiong YY, Chen GH, Qian HY, Jin C, Yu Y, Cheng K, Qian L, Yang YJ (2020). Atorvastatin enhances the therapeutic efficacy of mesenchymal stem cells-derived exosomes in acute myocardial infarction via up-regulating long non-coding RNA H19. Cardiovasc Res.

[22] Barile L, Moccetti T, Marban E, Vassalli G (2017). Roles of exosomes in cardioprotection. Eur Heart J.

[23] Saparov A, Ogay V, Nurgozhin T, Chen WCW, Mansurov N, Issabekova A, Zhakupova J (2017). Role of the immune system in cardiac tissue damage and repair following myocardial infarction. Inflamm Res.

