# Supplementary material for: Stem cell-derived exosomes in the treatment of acute myocardial infarction in preclinical animal models: a meta-analysis of randomized controlled trials
Source: Stem Cell Res Ther. 2022 Apr 8;13:151. doi: 10.1186/s13287-022-02833-z (PMC8994329; doi:10.1186/s13287-022-02833-z)
Supplement: Supplementary file 1 — Additional file 1. Search strategy. [file 13287_2022_2833_MOESM1_ESM.docx]

1、Pubmed：（456）

((((vesicle OR vesicles OR microvesicle OR microvesicles OR micro-vesicle OR micro-vesicles OR nanovesicle OR nanovesicles OR nano-vesicle OR nano-vesicles OR microparticle OR microparticles OR micro-particle OR micro-particles OR exovesicle OR exovesicles OR “platelet dust” OR “platelet dusts” OR “apoptotic body” OR “apoptotic bodies” OR exosome OR exosomes OR secretome OR secretomes OR dexosome OR dexosomes OR texosome OR texosomes OR epididymosome OR epididymosomes OR tolerosome OR tolerosomes OR prostasome OR prostasomes OR ectosome OR ectosomes OR “extracellular vesicles”[MeSH Terms] OR “cell-derived microparticles”[MeSH Terms] OR “exosomes”[MeSHTerms])) AND ("myocardial infarction" OR "myocardial infarctions" OR "myocardial infarct" OR "myocardial infarcts" OR "cardiac infarction" OR "cardiac infarctions" OR "ventricular infarction" OR "ventricular infarctions" OR "myocardium infarction" OR "heart infarction" OR "heart infarctions" OR "myocardial ischemia" OR "myocardial ischemias" OR "myocardial necrosis" OR "myocardial necroses" OR "myocardial reperfusion" OR "myocardial reperfusions" OR "myocardial remodel" OR "myocardial remodeling" OR "ventricular remodeling" OR "atrial remodeling" OR "heart attack" OR "heart attacks" OR "coronary occlusion" OR "coronary occlusions" OR "coronary ligation" OR "coronary ligations" OR “myocardial infarction”[MeSH Terms] OR “myocardial ischemia”[MeSH Terms] OR “myocardial reperfusion”[MeSH Terms] OR “ventricular remodeling”[MeSH Terms] OR “atrial remodeling”[MeSH Terms] OR “coronary occlusion”[MeSH Terms])) AND ((("stem cell"[MeSH Terms]))

2、Embase (817):

#3 (410137)

#2 (572081)

#1 (81882)

(vesicle OR vesicles OR microvesicle OR microvesicles OR 'micro vesicle' OR 'micro vesicles' OR nanovesicle OR nanovesicles OR 'nano vesicle' OR 'nano vesicles' OR microparticle OR microparticles OR 'micro particle' OR 'micro particles' OR exovesicle OR exovesicles OR 'platelet dust' OR 'platelet dusts' OR 'apoptotic body' OR 'apoptotic bodies' OR exosome OR exosomes OR secretome OR secretomes OR dexosome OR dexosomes OR texosome OR texosomes OR epididymosome OR epididymosomes OR tolerosome OR tolerosomes OR prostasome OR prostasomes OR ectosome OR ectosomes OR 'exosomes'/exp) AND ('myocardial infarction' OR 'myocardial infarctions' OR 'myocardial infarct' OR 'myocardial infarcts' OR 'cardiac infarction' OR 'cardiac infarctions' OR 'ventricular infarction' OR 'ventricular infarctions' OR 'myocardium infarction' OR 'heart infarction' OR 'heart infarctions' OR 'myocardial ischemia' OR 'myocardial ischemias' OR 'myocardial necrosis' OR 'myocardial necroses' OR 'myocardial reperfusion' OR 'myocardial reperfusions' OR 'myocardial remodel' OR 'myocardial remodeling' OR 'ventricular remodeling' OR 'atrial remodeling' OR 'heart attack' OR 'heart attacks' OR 'coronary occlusion' OR

'coronary occlusions' OR 'coronary ligation' OR 'coronary ligations' OR 'heart infarction'/exp OR 'heart muscle ischemia'/exp OR 'heart muscle reperfusion'/exp OR 'heart ventricle remodeling'/exp OR 'coronary artery occlusion'/exp) AND (stem cell)

3、Web of Science (392):

TOPIC: (vesicle OR vesicles OR microvesicle OR microvesicles OR micro-vesicle OR micro-vesicles OR nanovesicle OR nanovesicles OR nano-vesicle OR nano-vesicles OR microparticle OR microparticles OR micro-particle OR micro-particles OR exovesicle OR exovesicles OR “platelet dust” OR “platelet dusts” OR “apoptotic body” OR “apoptotic bodies” OR exosome OR exosomes OR secretome OR secretomes OR dexosome OR dexosomes OR texosome OR texosomes OR epididymosome OR epididymosomes OR tolerosome OR tolerosomes OR prostasome OR prostasomes OR ectosome OR ectosomes) AND

TOPIC: ("myocardial infarction" OR "myocardial infarctions" OR "myocardial infarct" OR "myocardial infarcts" OR "cardiac infarction" OR "cardiac infarctions" OR "ventricular infarction" OR "ventricular infarctions" OR "myocardium infarction" OR "heart infarction" OR "heart infarctions" OR "myocardial ischemia" OR "myocardial ischemias" OR "myocardial necrosis" OR "myocardial necroses" OR "myocardial reperfusion" OR "myocardial reperfusions" OR "myocardial remodel" OR "myocardial remodeling" OR "ventricular remodeling" OR "atrial remodeling" OR "heart attack" OR "heart attacks" OR "coronary occlusion" OR "coronary occlusions" OR "coronary ligation" OR "coronary ligations")

AND TOPIC: ("stem cell")

Indexes=SCI-EXPANDED Timespan=All years
