# Supplementary material for: Stem cell-derived exosomes in the treatment of acute myocardial infarction in preclinical animal models: a meta-analysis of randomized controlled trials
Source: Stem Cell Res Ther. 2022 Apr 8;13:151. doi: 10.1186/s13287-022-02833-z (PMC8994329; doi:10.1186/s13287-022-02833-z)
Supplement: Supplementary file 2 — Additional file 2. Quality of eligible studies. [file 13287_2022_2833_MOESM2_ESM.docx]

Quality of eligible studies.

| **Study** | **A** | **B** | **C** | **D** | **E** | **F** | **G** | **H** | **I** | **J** | **Total** |
| --- | --- | --- | --- | --- | --- | --- | --- | --- | --- | --- | --- |
| Changchen Xiao 2018 | **√** | **√** | **√** |  | **√** | **√** | **√** |  | **√** | **√** | 8 |
| Christopher J 2020 | **√** | **√** | **√** |  |  | **√** | **√** |  | **√** | **√** | 7 |
| CS Zhang 2019 | **√** | **√** | **√** |  |  | **√** | **√** |  | **√** | **√** | 7 |
| Hui Huang 2020 | **√** | **√** | **√** |  | **√** | **√** | **√** |  | **√** | **√** | 8 |
| LingSun 2020 | **√** | **√** | **√** |  | **√** | **√** | **√** |  | **√** | **√** | 8 |
| Peisen Huang 2019 | **√** | **√** | **√** |  | **√** | **√** | **√** |  | **√** | **√** | 8 |
| Xiaolin Liu 2020 | **√** | **√** | **√** |  |  | **√** | **√** |  | **√** | **√** | 7 |
| Xinlong Wang 2018 | **√** | **√** | **√** |  |  | **√** | **√** |  | **√** | **√** | 7 |
| Yong Li 2019 | **√** | **√** | **√** |  |  | **√** | **√** |  | **√** | **√** | 7 |
| Zilun Wei 2019 | **√** | **√** | **√** |  |  | **√** | **√** |  | **√** | **√** | 7 |

Abbreviation:

A peer-reviewed journal; B temperature control; C animals were randomly allocated; D blind established model; E blinded outcome assessment; F use of anesthetic without significant intrinsic vascular protection activity; **G** , appropriate animal model (diabetic, advanced age or hypertensive); **H** calculation of sample size; **I** statement of compliance with animal welfare regulations; **J** statement of potential conflict of interests.
