# Supplementary figures and images for: Stem cell-derived exosomes in the treatment of acute myocardial infarction in preclinical animal models: a meta-analysis of randomized controlled trials
Source: Stem Cell Res Ther. 2022 Apr 8;13:151. doi: 10.1186/s13287-022-02833-z (PMC8994329; doi:10.1186/s13287-022-02833-z)

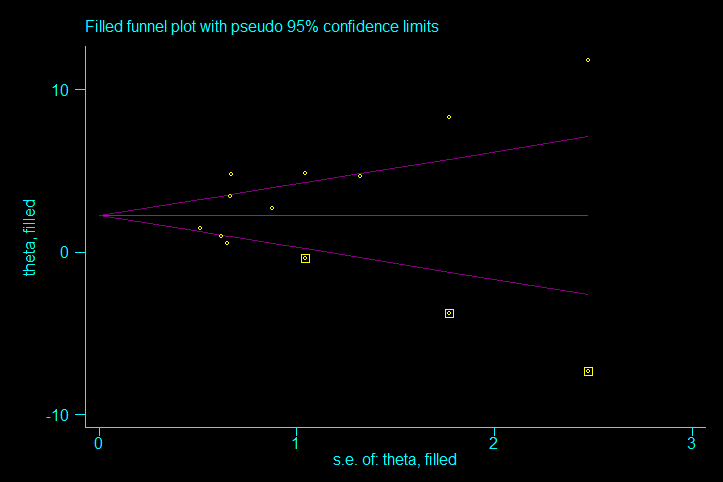

Supplement: Supplementary file 3 — Additional file 3. Trim-and-fill test. [file 13287_2022_2833_MOESM3_ESM.png]
